# Renal macrophages and NLRP3 inflammasomes in kidney diseases and therapeutics

**DOI:** 10.1038/s41420-024-01996-3

**Published:** 2024-05-13

**Authors:** Mohammad Islamuddin, Xuebin Qin

**Affiliations:** 1 Division of Comparative Pathology, Tulane National Primate Research Center, Tulane University School of Medicine, Tulane University, 18703 Three Rivers Road, Covington, LA 70433 USA; 2 Department of Microbiology and Immunology, School of Medicine, Tulane University, New Orleans, LA 70112 USA

**Keywords:** Inflammasome, Kidney diseases

## Abstract

Macrophages are exceptionally diversified cell types and perform unique features and functions when exposed to different stimuli within the specific microenvironment of various kidney diseases. In instances of kidney tissue necrosis or infection, specific patterns associated with damage or pathogens prompt the development of pro-inflammatory macrophages (M1). These M1 macrophages contribute to exacerbating tissue damage, inflammation, and eventual fibrosis. Conversely, anti-inflammatory macrophages (M2) arise in the same circumstances, contributing to kidney repair and regeneration processes. Impaired tissue repair causes fibrosis, and hence macrophages play a protective and pathogenic role. In response to harmful stimuli within the body, inflammasomes, complex assemblies of multiple proteins, assume a pivotal function in innate immunity. The initiation of inflammasomes triggers the activation of caspase 1, which in turn facilitates the maturation of cytokines, inflammation, and cell death. Macrophages in the kidneys possess the complete elements of the NLRP3 inflammasome, including NLRP3, ASC, and pro-caspase-1. When the NLRP3 inflammasomes are activated, it triggers the activation of caspase-1, resulting in the release of mature proinflammatory cytokines (IL)-1β and IL-18 and cleavage of Gasdermin D (GSDMD). This activation process therefore then induces pyroptosis, leading to renal inflammation, cell death, and renal dysfunction. The NLRP3–ASC–caspase-1–IL-1β–IL-18 pathway has been identified as a factor in the development of the pathophysiology of numerous kidney diseases. In this review, we explore current progress in understanding macrophage behavior concerning inflammation, injury, and fibrosis in kidneys. Emphasizing the pivotal role of activated macrophages in both the advancement and recovery phases of renal diseases, the article delves into potential strategies to modify macrophage functionality and it also discusses emerging approaches to selectively target NLRP3 inflammasomes and their signaling components within the kidney, aiming to facilitate the healing process in kidney diseases.

## Facts

- Several kidney diseases involve a significant involvement of both M1 and M2 macrophages.
- M1 macrophages contribute to renal tissue damage, inflammation, and fibrosis, while M2 macrophages play a role in tissue repair and regeneration.
- Renal macrophages possess all components of NLRP3 inflammasomes, which, upon activation, produce proinflammatory cytokines cleave GSDMD, and induce pyroptosis.
- Exploring the modulation of macrophage polarization, NLRP3 inflammasomes, and their signaling components could unveil potential therapeutic agents against kidney disease.

## Questions

- The origin and differentiation of renal macrophages into M1 or M2 phenotypes, and vice versa, during renal disease conditions are yet to be fully understood.
- How does NLRP3 inflammasome activation in renal macrophages play a major role in different kidney diseases?
- Can selectively targeting macrophage polarization and NLRP3 inflammasomes and its signaling components lead to effective treatment in kidney disease?

## Introduction

Macrophages form a diverse group of cells within the mononuclear phagocyte system, crucially contributing to homeostasis, remodeling, and immune regulation in the development of kidney disease pathogenesis. They represent a promising target for therapeutic interventions aimed at addressing kidney injury and fibrosis [1]. The kidney injury and fibrosis process involves chronic inflammation and unsuccessful injury repair by the macrophages [2]. Macrophages found in kidney tissue might come from various sources like erythromyeloid progenitors, hematopoietic stem cells, or circulating monocytes throughout different developmental stages and in response to the tissue injury [3]. These resident macrophages can exhibit different phenotypes, such as classically activated macrophages (M1) and alternatively activated macrophages (M2), influenced by the specific microenvironment of the tissue [4, 5]. M1 macrophages after induction with IFN-g and LPS secrete proinflammatory cytokines and are considered as proinflammatory macrophages and accelerate the process of kidney injury, while M2 macrophages play an anti-inflammatory role and could be additionally sub-divided toward M2a, M2b, & M2c, these macrophages induced anti-inflammation, wound healing, immune regulation, tissue regeneration and fibrosis [1, 6], TGF-b secreted by the M2 macrophages sub-types play a very essential function in myofibroblast differentiation and extracellular matrix (ECM) accumulation via TGF-β/Smad signaling pathway. In the kidney, resident mononuclear cells like macrophages and dendritic cells possess complete inflammasomes and are susceptible to activation. This susceptibility arises from continuous exposure to diverse DAMPs (Damage-Associated Molecular Patterns) and PAMPs (Pathogen-Associated Molecular Patterns) [7, 8]. Inflammasomes are elements of the body’s natural defense mechanism (Innate immune system) that react to cellular stress by activating caspase 1. Activation of caspase-1 generate proinflammatory signaling molecules interleukin (IL)-1β and IL-18. Additionally, caspase 1 cleaves the Gasdermin D (GSDMD) and performs a role in facilitating a type of inflammatory cell death known as pyroptosis [9]. The activation of inflammasomes causing inflammation is associated with the onset of acute kidney injury, fibrosis, and chronic kidney disease. Among the various inflammasomes, the NLRP3 (NOD-like receptor family, pyrin-containing domain-3) inflammasome is extensively studied and has been involved in kidney-related disorders [8, 10]. The NLRP3 inflammasome is essential in facilitating the maturation and discharge of proinflammatory cytokines, triggering heightened inflammatory responses that result in irreversible body injury [11]. Numerous investigations have additionally demonstrated the involvement of the NLRP3 inflammasome in the progression of chronic kidney diseases (CKD) [12–15]. This review offers a comprehensive overview of kidney macrophages, covering aspects such as homeostasis, plasticity, and regulation, along with exploring the functions of different phenotypes, and examines the involvement of NLRP3 inflammasomes in renal damage, inflammation, and fibrosis. It aims to enhance comprehension of changes in the kidney microenvironment, factors influencing macrophage phenotype and functions, and potential therapeutic targets for kidney diseases.

## Kidney residential macrophages origin

**Fig. 1:**
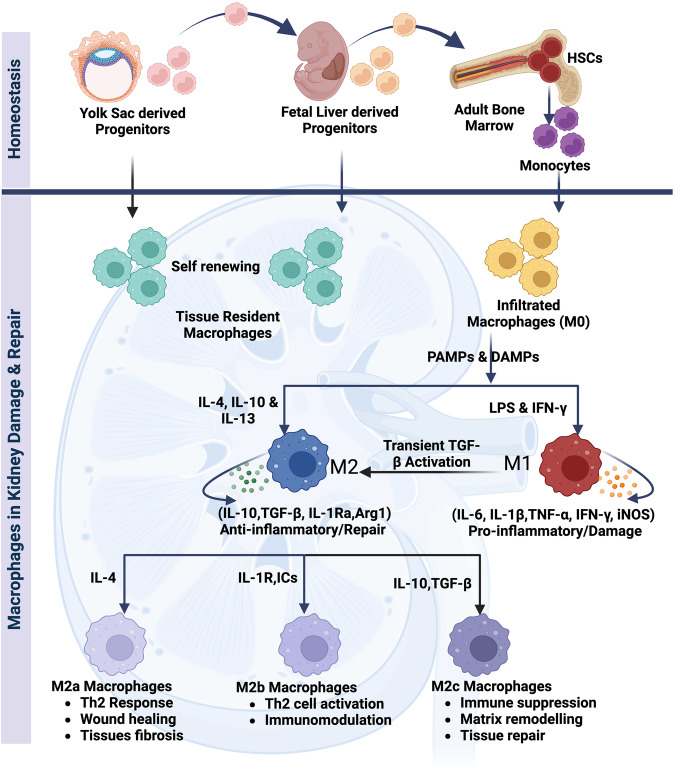
Macrophages are seeded into renal tissues at various embryonic stages, originating from three distinct sources: the yolk sac, fetal liver, and bone marrow. These developmental phases give rise to tissue-resident macrophage populations, which possess self-renewal capabilities and contribute to anti-inflammatory processes during kidney repair. The damage inflicted by pathogen-associated molecular patterns (PAMP) and damage-associated molecular patterns (DAMP), as well as inflammatory cytokines, triggers the activation and differentiation of M1 and M2 macrophages. M1 macrophages, induced by cytokines IFN-g and LPS, exhibit cytotoxic and inflammatory functions, releasing IL-1β, TNF-α, IFN-γ, IL-6, and iNOS, thereby promoting renal injury. On the other hand, M2 macrophages, polarized by IL-4 and IL-10, produce anti-inflammatory cytokines like IL-10, TGF-β, and IL-1Ra, expediting the resolution of inflammation and facilitating the repair process. In the tissue repair phase, M2b macrophages modulate immunity, while M2c macrophages suppress immune responses, engage in matrix remodeling, and contribute to tissue repair. However, if inflammation persists and wound healing processes are uncontrolled, renal fibrosis may ensue. This occurs through the activation and differentiation of macrophages into M2a subtypes in the injured kidney, driven by IL-4, leading to increased production of transforming growth factor b (TGF-β) and eventual kidney fibrosis. Figure created using BioRender.com.

Macrophages, which are innate immune cells with phagocytic abilities, are crucial for defending the host and maintaining tissue equilibrium. The understanding of macrophage biology has significantly advanced in recent years, revealing that tissue-resident macrophages exhibit greater complexity and diversity than previously recognized. Macrophages residing in the kidneys undergo self-renewal on-site and are identified by their phagocytosis ability, the presence of pattern recognition receptors (PRRs), and their capacity for immunological regulation. In this way, they play a crucial role in preserving kidney homeostasis, participating in both tissue injury and tissue-repair processes [3, 16–18]. One distinguishing feature of most immune cells involves their regular replenishment originating from precursors, these precursors are hematopoietic stem cells (HSCs) that come from the bone marrow [19]. In the kidney, all myeloid cells, which encompass granulocytes (such as eosinophils, basophils, neutrophils, and mast cells), monocytes, and dendritic cells, originate from HSCs, with the exception of tissue-resident macrophages [20, 21]. Kidney-resident macrophages have three distinct sources: (1) macrophages derived from yolk sac erythro-myeloid progenitors (EMP), (2) macrophages derived from fetal liver EMP, and (3) macrophages derived from hematopoietic stem cells (HSC) [18, 22, 23]. The distribution of each progenitor cell type undergoes significant shifts throughout the stages of development, adulthood, and when the kidney is in a damaged state [24]. From an ontological perspective, it has been suggested that the mononuclear phagocytic system (MPS) originates from a strict chronological sequence of macrophage progenitors [25]. In mice, the initial development of macrophages begins at day 8 of embryogenesis within the primitive ectoderm of the yolk sac. These macrophages originate without a monocytic progenitor, as indicated by Liu et al. [18]. This primitive process is succeeded by definitive hematopoiesis within fetus liver. At first, the fetal liver receives hematopoietic progenitors from the yolk sac and and then afterward from the hematogenic endothelium of aortogonadal mesonephros region of the embryo. As embryogenesis progresses, the fetal liver becomes the primary source of definitive hematopoiesis, giving rise to circulating monocytes, including resident, lymphocyte antigen 6c negative (Ly6c2), and inflammatory Ly6c1 monocytes [26]. Postnatally, as bone formation takes place, fetal liver hematopoiesis diminishes, and bone marrow hematopoiesis takes precedence. This definitive hematopoiesis serves as the origin of circulating monocytes in mice. It has been suggested that all tissue-resident macrophages trace their origins back to this definitive hematopoiesis phase [27] (Fig. 1**)**. A thorough examination of mononuclear phagocytes in the kidneys of residents has recognized five different populations, mainly distinguished by the surface expression of CD11b and CD11c. This classification further takes into account the surface marker of F4/80, CD103, CD14, CD16, and CD64 [28]. Understanding the source of tissue resident macrophages and the dynamics involving in situ proliferation and replacement by monocytes continues to be a crucial area of study. Examination of the transcriptional profiles of resident tissue macrophages has uncovered distinct transcriptional programs specific to each tissue [19, 29]. These findings imply that tissue macrophages possess inherent programming tailored to address the unique requirements of their residing tissues. The increasing awareness of the in vivo diversity among tissue macrophages highlights the importance of redefining states of macrophage activation.

## Kidney macrophage heterogeneity

The diverse nature of macrophages has been a subject of interest since their discovery. Under normal physiological conditions, macrophages exhibit unique characteristics, and in the presence of factors like Hypoxia, cellular damage, pathogens, immune responses, and tissue repair, they undergo differentiation to fulfill specific functions. In instances of heightened recruitment during diseases, inflammatory monocytes respond to cytokine signals, giving rise to two distinct macrophage subsets known as M1 and M2 [30]. M1 macrophages are identified by their pro-inflammatory effects and interaction with T helper 1 (Th1) cells. On the other hand, M2 macrophages display immunoregulatory actions and closely collaborate with T helper 2 (Th2) cells [5, 31–34]. M1 macrophages perform a vital role in host defense by participating in a Th1-like immune response [1]. The polarization of M1 macrophages could be initiated through various stimuli, including PAMPs like lipopolysaccharide (LPS), DAMPs or alarmins such as S100A9 and IL-1α, pro-inflammatory cytokines like IFNγ and tumor necrosis factor (TNF) [35–39]. This M1 polarization is characterized by the elevated levels of proinflammatory cytokines such as IL-6, IL-1, IL-23, IL-12, matrix metalloproteinase 12 (MMP12), iNOS and MINCLE [40–43]. Monocytes that infiltrate damaged tissue have the capacity to undergo alternative activation, adopting an M2 phenotype [1]. In contrast to its traditionally activated counterpart, the M2 macrophage plays a role in resolving inflammation by possessing robust endocytic clearance abilities and generating trophic factors. This results in a decreased secretion of proinflammatory cytokines [44, 45]. Specifically, M2 macrophages release trophic factors that stimulate angiogenesis and facilitate the healing of wounds by modifying the extracellular matrix (ECM) [46]. Additionally, M2 macrophages express fibronectin 1 (FN-1), BIG-H3 (a matrix-associated protein induced by TGF-β), and IGF-1, all of which transmit signals for the proliferation and repairing of tissue [40, 47]. These cells produce arginase-1, which suppresses inflammation by hindering the formation of proinflammatory nitric oxide (NO) [46]. Moreover, M2 cells also expressed IL-1R antagonists and hence counteract the action of the proinflammatory cytokine IL-1, as well as the chitinase 3–like 3 (Ym-1) and the mannose receptor [48, 49]. These M2 macrophages could be categorized into three sub-groups such as M2a, M2b, and M2c based on their phenotype and functionality. Different stimuli drive macrophage polarization into M2 phenotypes: M2a macrophage differentiation induced by IL-4 and IL-13 cytokines [35, 50], TLR and/or IL-1R ligands and immune complexes induce M2b macrophage differentiation [51, 52] conversely IL-10, TGF-β, and glucocorticoids leads to activate M2c macrophage differentiation [53]. The various sub-types of M2 macrophages exhibit both unique and overlapping roles [44]. M2a macrophages, for instance, stimulate TH2-like anti-inflammatory immune response, fostering both healing of wound and tissue fibrosis. Macrophages sub-type M2b engage regulation of the immune system and play a role in TH2-like activation. On the other hand, M2c sub-type macrophages are involved in immunosuppression, participate in the remodeling of the matrix, and aid in tissue repair [44, 54–57] Fig. 1.

## Kidney injury and M1 macrophages

A variety of different stimuli can trigger the development of an M1 proinflammatory macrophage phenotype within a damaged kidney. Cytokines and chemotactic factors present in the pathological microenvironment of diseased kidneys recruit circulating monocytes. These monocytes accumulate on activated endothelial surfaces, infiltrate both glomerular and tubulointerstitial sections, and transform into pro-inflammatory M1 macrophages [1]. The extent of macrophage inflation is closely linked to the severity of renal impairment, increased cell density in the glomeruli, segmental lesions, and crescent formation observed in proliferative types of glomerulonephritis [58], as well as in scenarios involving the rejection of renal allograft in transplantation models [59]. Classically activated macrophages M1 macrophages, exhibit functional traits that are both pro-inflammatory and antimicrobial. The polarization of the M1 macrophages is induced by PAMPs like alarmins (S100A9), IFN-γ and/or LPS, TNF-α and IL-1a [35, 36, 39, 40]. Chemokines and Proinflammatory cytokines, including IL-6, TNF-α, IL-12, IL-1, IL-8, and nitric oxide, are secreted by M1 macrophages, contributing to inflammation and tissue damage [1, 40, 60] (Fig. 1). In rat models during kidney ischemia-reperfusion injury (IRI), macrophages undergo polarization to the M1 (classically activated) phenotype and exhibit substantial expression of iNOS [28, 61]. The activation of M1 macrophage, exacerbated kidney injury in acute forms as well as in progressive forms of kidney disease models. The activation is triggered by the release of endogenous DAMPs such as DNA and high mobility group protein B1 (HMGB1) from damaged and necrotic cells, along with advanced glycation end-products and C-reactive protein [62–65]. The introduction of bacterial (CpG) DNA or LPS in kidney disease models intensifies the macrophage M1 response, exacerbating renal damage [66, 67]. MINCLE, a transmembrane PRR, is specifically present in macrophages within the kidney and is activated during the early phases of both UUO and cisplatin-induced AKI. Lv et al. established a direct association between macrophages expressing Mincle and AKI. Their research showed that transferring macrophages with suppressed Mincle expression had a protective effect on the kidney in the cisplatin-induced AKI model [42].

Adoptive transfer of M1 macrophages stimulated with IFNγ exacerbates glomerular damage more than transferring unstimulated M1 macrophages in acute anti-glomerular basement membrane disease models [68]. Similarly, in adriamycin nephrosis models, adoptive transfer of M1 macrophages stimulated with LPS results in significantly highly severe kidney injury compared to the transfer of unstimulated macrophages [45]. One of the characteristic features of M1 macrophages is the activation of NF-κB signaling, which contributes to kidney damage in rats with anti-glomerular basement membrane disease [69]. In contrast, macrophages with inhibited NF-κB activation exhibit an anti-inflammatory phenotype and mitigate kidney damage upon adoptive transfer [70], these findings underscore the pivotal role of NF-κB in the kidney damage caused by M1 macrophages. The reduction of macrophages through the use of liposome clodronate significantly lessened renal damage, accompanied by a decrease in the formation of inflammatory and profibrotic cytokines [71]. Similarly, the administration of miR-30c-5p agomir, which directly suppresses Interferon regulatory factor 1 (IRF1), resulted in a decrease in ischemic renal injury by lowering M1 macrophages levels and elevating the level of M2 macrophages. This was achieved through a reduction in the inflammatory cytokine TNF-α and an increase in anti-inflammatory cytokines IL-4 and IL-10 [72, 73]. Conversely, the infusion of IFN-induced M1 macrophages after acute kidney ischemia-reperfusion injury led to an elevation in tubulointerstitial fibrosis and functional detriment [34]. Aldosterone, primarily recognized for its pivotal role in maintaining electrolyte balance and blood pressure via actions in the kidney, exerts influence over macrophage function by modulating gene expression patterns. Several studies indicate that aldosterone could stimulate inflammation by increasing the expression of pro-inflammatory genes in macrophages [74–77]. Additionally, aldosterone’s impact on interstitial salt gradients indirectly affects macrophage function by modifying the osmotic environment and ion concentrations in the tissue. This alteration can impact various aspects of macrophage behavior including migration, activation, and cytokine production, thus influencing immune responses and inflammatory processes [78]. Therefore, aldosterone plays a role in the polarization of M1 macrophages and affects the extent of kidney damage in experimental models. An M1 phenotype in macrophages prompted by Aldosterone [79], while the conditional knock out of its receptor, the mineralocorticoid receptor, in myeloid cells inhibits polarization of M1 macrophage, leading to reduced kidney damage in crescentic glomerulonephritis mouse models [80]. Other molecules such as miR-146 play a pivotal role in macrophage polarization, whereby its dysregulation can lead to kidney damage. Specifically, miR-146a demonstrates the ability to inhibit the pro-inflammatory M1 phenotype, while its absence in diabetic mice exacerbates the M1 response and attenuates the M2 response, thereby exacerbating kidney injury. In the context of renal inflammation, M1 macrophages have the potential to speed up the generation of inflammatory cytokines like IL-1β and or TNF-α, leading to the initiation of renal injury. Apart from these inflammatory cytokines, macrophages in an activated state release matrix metalloproteinase (MMPs), and these MMPs break down the extracellular matrix and consequently contribute to both matrix degradation and inflammatory damage within the kidney [81, 82]. Macrophage-derived MMP-9 plays a role in kidney fibrosis by inducing pro-fibrotic alterations in tubular epithelial cells [83]. Importantly, the proteolytic release and activation of TGF-β, which is sequestered within the extracellular matrix and facilitated by MMPs [84], could also contribute to renal fibrosis. When M1 macrophages are stimulated with IL-1 and LPS, they generate MMP12 [85]. Genetic or pharmacological blockade of MMP12 has been demonstrated to reduce both macrophage infiltration and activation, thereby preventing crescent formation and alleviating severe glomerular damage [86].

## Renal fibrosis and M2 macrophages

**Fig. 2:**
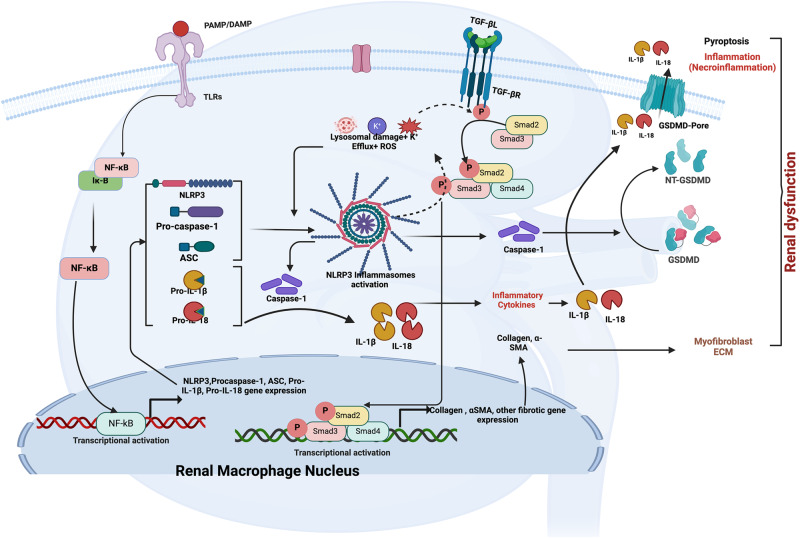
A diagrammatic illustration of NLRP3 inflammasomes and TGF-β-induced kidney fibrosis is depicted as follows. When danger signals like damage-associated (DAMP) and pathogen-associated molecular patterns (PAMP) bind to Toll-like receptors (TLRs) on renal macrophages/dendritic cells, it triggers the transcriptional activation of NF-κB signaling. This activation results in an increased expression of components associated with inflammasomes, such as NLRP3, ASC, Procaspase-1, Pro-IL-1β, Pro-IL-18, and others. Consequently, events like potassium efflux (*K* + ), reactive oxygen species (ROS) generation, and lysosomal damage are induced. These events lead to the activation and oligomerization of NLRP3, which then recruits ASC and procaspase-1 to form inflammasome complexes, ultimately activating procaspase-1 into bioactive caspase-1. Activated caspase-1 plays a role in cleaving Pro-IL-1β and Pro-IL-18 into mature IL-1β and IL-18 inflammatory cytokines, respectively, contributing to the mediation of inflammation. Additionally, activated caspase-1 cleaves GSDMD to NT-GSDMD, which induces pore formation in the plasma membrane and mediates pyroptosis, a regulated form of necrosis (Necroinflammation). Simultaneously, TGF-β binds to TGF-β receptors (TGF-βR), the activated NLRP3 inflammasomes induce the ROS generation and this ROS augments the phosphorylation of regulatory Smad proteins (Smad2, Smad3, and Smad4) and subsequent transcriptional activation of Smad2, Smad3, and Smad4. This activation promotes the expression of profibrotic genes such as collagen and aSMA. The interplay between the NF-κB/NLRP3/IL-1β/IL-18 axis and the TGF-β/Smad signaling pathway may contribute to the development of kidney fibrosis and associated damage. Figure created using BioRender.com.

Alternatively, activated M2 macrophages can be categorized into three functional subtypes based on in vitro experiments. These M2 macrophage subtypes are believed to play a role in dampening immune reactions and facilitating tissue regeneration, with divergent and occasionally controversial roles [87]. Macrophages sub-type M2a, characterized by high expression of the marker arginase 1 (Arg-1), generate significant quantities of anti-inflammatory cytokines such as IL-1 receptor antagonist (IL-1ra) and IL-10. Additionally, they inhibit the expression of proinflammatory cytokines like TNF-α, IL-12, and IL-1, as well as the production of nitric oxide, so that driving anti-inflammatory and immunosuppressive effects [40]. M2b macrophages, on the contrary, specifically increase the expression of IL-10 while decreasing IL-12. They activate T cells to release IL-4, which subsequently stimulates B cells for the production of antibodies, fostering an anti-inflammatory Th2 immune reaction. Lastly, the macrophages sub-types M2c release substantial quantities of TGF-β and IL-10, actively suppressing pro-inflammatory immune reactions and promoting the healing of wound and tissue fibrosis [1, 30, 44, 57]. The increase in population of M2c macrophage sub-types, characterized by the presence of CD206 and/or CD163 expression, correlatates with renal fibrosis in human kidney disease, as demonstrated by studies conducted by [88] Hu et al. and [89] Ikezumi et al. In individuals with diabetic renal disease, the quantity of macrophages expressing CD163 in the glomeruli is linked to glomerulosclerosis, interstitial fibrosis, and tubular atrophy, as revealed by research conducted by [90, 91] Klessens et al and Wu et al. Moreover, elevated levels of M2 macrophages expressing CD206 + and CD163 + have been identified in the peritoneal drainage of dialysis patients with peritonitis. In this context, the generation of CCL18 by M2 macrophages is associated with the gradual deterioration of ultrafiltration and the development of peritoneal fibrosis, as documented by [92] Bellon et al. The clinical observations strongly indicate the pro-fibrotic function of a distinct subset of M2 macrophages (M2c), underscoring the significance of understanding the role of macrophages in renal fibrosis. Together, these results suggest that the polarization and infiltration of M2c macrophages may play a role in kidney fibrosis and the progression of renal disease. Evidence advocating this assertion consists of findings that a decrease in the macrophage infiltration, particularly the M2 subtype, in mouse models of kidney disease may stop the progressive deposition of interstitial collagen and impede kidney fibrosis, as demonstrated by [53] Kim et al. Additionally, it was observed that the introduction of M2c macrophages, as opposed to M1 macrophages, resulted in a reversal of the beneficial effects associated with depleting macrophages in the context of renal fibrosis [1]. Similarly, the reduction of macrophages starting from day 4 in a UUO model significantly decreased kidney fibrosis. Conversely, the introduction of M2 macrophages through adoptive transfer enhanced fibrosis by causing the accumulation of αSMA+ cells [56, 93]. Canonical TGF-β signaling operates through TGF-βR1 and involves complexes such as Smad2, Smad3, and Smad4. TGF-β binds to TGF-βR, leading to the phosphorylation of regulatory Smad proteins (Smad2, Smad3, and Smad4) and then induction of transcriptional activation of Smad2, Smad3 & Smad 4, which leads to promoting the expression of the profibrotic gene (Fig. 2). TGFβ1 plays an important role in both inhibiting inflammation and facilitating tissue repair. However, paradoxically, it has been demonstrated by studies that it can also contribute to the progression of chronic fibrotic diseases [91, 94–96]. In the evolving stages of human kidney disease, there is an observed increase in both the expression and activation of TGFβ1 [97, 98]. Additionally, TGFβ1 serves as a significant inducer of M2 macrophage polarization [99, 100] and facilitates the differentiation and accumulation of myofibroblasts in the fibrotic kidney. This is evidenced by a decrease in the number of myofibroblasts when Tgf-βr2 (which encodes TGFβ receptor 2) is selectively removed in αSMA+ cells [101]. The pro-fibrotic consequence of TGF-β1 was extinct through a marked decrease in bone marrow-derived macrophages expressing GFP + , F4/80 + , and a-SMA + , as they underwent macrophage myofibroblast transformation (MMT), as demonstrated by [102] Wang et al. Consequently, renal fibrosis was diminished in the mouse study subjected to unilateral ureteral obstruction (UUO) [103].

## Macrophages (M1/M2) plasticity and regulation in pre-clinical research

Different subsets of macrophages can coexist within kidney tissue, with specific subsets prevailing at different disease stages, ranging from the onset of kidney injury to the recovery phase. The ability of macrophages to adapt their function in response to local microenvironmental cues during injury, inflammation, fibrosis, and repair underscores their phenotypic plasticity [104]. Numerous studies have linked macrophage accumulation in the kidneys to renal function, inflammation, and cellular damage. However, current research suggests that macrophages exhibit more complex phenotypes. In the early phases of kidney injury, macrophages residing in kidney, endowed with self-maintenance capabilities through proliferation [105], undergo activation triggered by PAMPs, DAMPs [106], IFN-γ, and inflammatory cytokines. This activation leads to their transformation into pro-inflammatory M1 macrophages, a phenotype typically associated with infection or cellular damage. Concurrently, circulating monocytes are recruited to the kidney, where they also differentiate into M1 macrophages. Thus, alterations in the kidney microenvironment significantly influence both the activation and infiltration of macrophages within diseased kidneys [104]. Macrophages possess the capability to acquire specific characteristics in response to particular stimuli. However, it remains uncertain whether these same cells can subsequently undergo further changes in response to alterations in their surrounding environment. This uncertainty is particularly relevant in conditions where a distinct transition from an M1 to an M2 phenotype occurs, as observed in renal IRI. In the initial stages of this injury, macrophages predominantly exhibit an M1 phenotype, while during the phase of tissue repair, they tend to predominantly display an M2 phenotype [1, 28, 34, 107]. Whether the transition from M1 to M2 phenotype involves a transformation within the same macrophage population or if distinct cell populations are involved remains unclear. A similar transition from an M1 to an M2 phenotype is also noted in models of glomerular disease. For example, in a rat model of nephrotoxic serum nephritis, macrophage infiltration primarily consists of M1 phenotype cells increase levels of NOS2 and MMP12 expression until day 14, coinciding with the peak of glomerular inflammation and cellular crescent formation. However, as glomerular and interstitial fibrosis progresses, there is a simultaneous reduction in macrophage numbers, accompanied by a shift towards an anti-inflammatory M2 macrophages characterized by decreased levels of NOS2 and MMP12 expression and increased levels of CD163 and CD206 expression [108]. The compromised recovery of the kidney noted after macrophage depletion during the tubular proliferative phase post-ischemia/reperfusion (I/R) injury suggests the essential role of macrophages present within the kidney at this stage for normal tubular repair. Additionally, these macrophages represent a unique subset in terms of their phenotypes compared to those present during the early reperfusion phase. An extensive analysis of macrophage populations, isolated based on the differential expression of Ly6C in CD11b + cells in the kidney post-I/R injury [109, 110], supported earlier findings, suggested that macrophages in the kidney shortly after reperfusion, predominantly characterized by CD11b + /Ly6C high cells, exhibit genes related to inflammation. Conversely, macrophages engaged in the tubular repair phase, identified by CD11b + /Ly6Cint cells, display markers associated with wound healing [34]. Ultimately, the phenotype and function of macrophages govern the course of inflammation and the formation of irreversible tissue scarring. Studies have demonstrated that non-specific removal of macrophages, accomplished through the use of anti-macrophage serum or liposomal clodronate, can attenuate experimental acute kidney injury by interrupting sustained inflammation and the subsequent development of fibrosis [71, 111, 112]. Complete depletion of macrophages via sublethal irradiation prevents their infiltration into injured kidneys and reduces the severity of fibrosis [113, 114]. Numerous studies indicate that even under suppressed inflammation, the presence of M2 macrophages doesn’t consistently correlate with fibrosis, regardless of disease duration. However, contrasting observations suggest that while eliminating inflammatory M1 macrophages doesn’t necessarily shield against kidney fibrosis, depleting anti-inflammatory and reparative M2 macrophages can alleviate it [115]. These contradictory findings may imply that the timing of macrophage depletion could be crucial in strategies aimed at promoting renal repair. Additionally, depleting one macrophage type might prompt a shift toward another due to altered microenvironments in diseased tissues [116]. Hence, rather than targeting specific macrophage subtypes, it may be more effective to address the altered microenvironment in diseased tissues. Elevated levels of pro-inflammatory cytokines, such as IL-4 and IL-13, hinder recovery from I/R-induced AKI, leading to increased M1 marker expression and decreased expression levels of M2 markers [50]. Evidence indicates that blocking the initial influx of macrophages mitigates I/R-induced renal injury [117]. Early depletion of macrophages prompts renal damage remission, whereas later depletion impairs recovery, underscoring the critical role of macrophage polarization during I/R-induced AKI recovery [34]. Recent studies also reveal the interplay between tubular epithelium and interstitial cells, where damaged tubular epithelial cells engage with macrophages during the repair and regeneration of acute kidney injury and disease advancement [118, 119]. Conversely, injured tubular epithelial cells can activate M1 macrophages in renal injury [120]. Therefore, maintaining cellular homeostasis of macrophage polarization may promote tubular epithelial cell population and kidney recovery after ischemia/reperfusion injury. Tribbles homolog 1, an adapter protein influencing immune-related transcription factor protein degradation, plays a critical role in macrophage differentiation. Depletion of Trib1 increases the neutrophil population but lower the M2 macrophage population [121]. Tribbles homolog 1 also regulates kidney recovery and regeneration by modulating renal tubular cell proliferation through M1/M2 macrophage polarization [122].

## Macrophages (M1/M2) plasticity and regulation in clinical research

The shift of macrophages from an M1 to an M2 phenotype is well-documented in animal models of kidney disease. However, in human kidney disease, this transition is less clearly understood due to the lack of repeated biopsies and the complicating effects of immunotherapies that directly affect macrophages. AKI poses a serious health threat with heightened morbidity and mortality rates, and a significant number of survivors progress to chronic kidney disease (CKD) [123–126]. Macrophage proliferation and polarization are essential for AKI recovery [127]. While macrophage polarization towards the M2 phenotype can alleviate inflammation, it may also contribute to tissue fibrosis. Particularly, the increased presence of M2 macrophages assumes a pivotal role in the transition from AKI to CKD in humans, as these macrophages possess the potential to facilitate this transition [128]. This occurrence might be linked to the expression of transforming growth factor-β (TGF-β), a pro-fibrotic factor, by M2 macrophages [55]. In the section discussing the AKI-CKD transition, deficiency of IRF4 impedes the AKI-CKD transition by restraining macrophage-to-fibroblast transformation, inhibiting macrophage M1-M2 polarization, and reducing inward neutrophil flow [129]. The crucial involvement of endothelin-1 in tissue fibrosis has been extensively established [130]. Administration of R-715 has been demonstrated to elevate endothelin-1 levels, resulting in heightened renal fibrosis. Budu et al, observed that deletion and antagonism of B1R did not lead to increased macrophages, thereby preventing the transition from M1 to M2 polarization [131]. Although this intervention offers acute protection, it may induce maladaptive tubular regeneration and mild toxicity, exacerbating renal fibrosis. Antibody-mediated rejection is a primary cause of allograft dysfunction and loss [132]. In both acute and chronic cases of antibody-mediated rejection, macrophage infiltration is common, yet their functions vary among different macrophage subsets [34]. Assessing the clinicopathologic impact of macrophage polarization in renal allograft patients with antibody-mediated rejection, [133] observed that individuals with glomerular M2 polarization exhibited elevated chronic glomerulonephritis scores, likely attributable to chronic glomerular injury. Conversely, tubulointerstitial M1 polarization correlated with heightened microvascular inflammation and intimal arteritis, supporting the proinflammatory role of M1 macrophages [34, 134]. Glomerular M2 polarization correlated with poorer graft function and tended to result in shorter graft survival, whereas individuals with tubulointerstitial M2 polarization showed no significant differences in renal function and graft survival compared to those with tubulointerstitial M1 polarization. In biopsy samples from cases of antibody-mediated rejection, glomerular M2 polarization was associated with chronic glomerular injury and poorer graft function, though not with graft survival [133]. These findings suggest that polarization of macrophages might offer a pathway for discovering new biomarkers and devising efficient therapeutic targets [135]. Classic markers for M1 macrophages encompass MHC class II (HLA-DR), CD80/CD86, and IL-1R, whereas those for M2 macrophages include mannose receptor (CD204, CD206), scavenger receptor (CD163), and CD23 [109, 136]. Recent studies indicate that the population of CD163 + M2 macrophages are prevalent in renal biopsies of proliferative glomerulonephritis, such as lupus nephritis, anti-neutrophil cytoplasmic antibodies (ANCA)-associated pauci-immune necrotizing glomerulonephritis, and membranoproliferative glomerulonephritis. Urinary soluble CD163 levels reflect glomerular inflammation in these disease conditions [137, 138]. In cases of proliferative glomerulonephritis and acute tubulointerstitial nephritis, CD163 + M2 macrophages were found to be the most abundant subtype in both the glomerular and interstitial compartments. However, their pathological significance differed among various conditions [139–141]. CD163 + M2 macrophages were associated with the extent of glomerular damage, suggesting their potential involvement in the acute inflammatory injury to glomeruli. Conversely, in the interstitial area, HLA-DR + M1 macrophages were correlated with the severity of acute tubulointerstitial injury, tubulitis, and dysfunction of renal tubules, whereas CD163 + M2 macrophages were more commonly linked with interstitial fibrosis [137, 138, 142–144]. Abundant M2 macrophages were observed in the kidneys of individuals with autosomal dominant polycystic kidney disease (ADPKD) or autosomal recessive polycystic kidney disease (ARPKD). These macrophages, identified by the M2 marker CD163, were discovered to stimulate proliferation and the formation of microcysts in vitro in ADPKD cyst cells [145]. In laboratory studies using human macrophages, it was found that an increase in CD163 expression resulted in a change in the secretion profile of cytokines from those associated with pro-inflammatory M1 responses to M2 cytokines [146, 147]. Macrophages expressing both CD163 and CD68 may play a role in the development of proliferative glomerular crescents, as seen in conditions like ANCA-associated glomerulonephritis or active lupus nephritis. These macrophages were also associated with proteinuria and estimated glomerular filtration rate (eGFR) [139]. Additionally, distinct populations of M2 macrophages, characterized by CD163 + and CD206 + markers, were predominantly found in fibrous crescents and were more prevalent in lupus nephritis (LN) and ANCA-associated vasculitis compared to IgA nephropathy and Henoch Schönlein purpura glomerulonephritis [88, 140]. Individuals in the early stages of idiopathic membranous nephropathy exhibited elevated levels of circulating CD14 + /CD163 + , CD14 + /CD163 + /CD206 + , and CD14 + /CD163 + /CD206 + /CD115+ macrophages compared to healthy controls [148]. M2 macrophages were identified as the primary subpopulation in human LN, with M2a subpopulations being linked to disease progression [149, 150]. In conditions like vascular or diabetic nephropathy (DN), chronic kidney diseases exhibit activation of glomerular macrophages, and the presence of glomerular anti-inflammatory CD163 + M2 macrophages correlates with pathological DN lesions [90, 151]. Studies on kidney biopsies from individuals with type 2 diabetes revealed a transient increase in the number of macrophages in the glomeruli during moderate glomerulosclerosis, which remained low during mild and advanced stages [152]. The balance between M1 and M2 macrophages shifts dynamically throughout the progression of DN at various stages [12, 153].

## Renal injury and inflammasomes

Innate immune response relies significantly on inflammasomes, which are crucial in reacting to both foreign microorganisms and internal danger signals, such as substances released by dying cells [154]. These complex assemblies of multiple proteins act as hubs for triggering caspase activity, which in turn controls the maturation of cytokines, inflammation, and cell death. Acute kidney injury (AKI) poses a significant global health challenge, with ~13.3 million diagnoses and 1.7 million associated deaths annually [155]. Renal cell necrosis is a key feature of acute kidney injury (AKI), commonly seen in conditions like thrombotic microangiopathies, necrotizing glomerulonephritis, or tubular necrosis. These conditions are characterized by intense inflammation within the kidneys, resulting in the loss of renal cells, a process referred to as necroinflammation [156]. Notably, in AKI, the inflammatory response is triggered by necroptosis rather than apoptosis [157]. When proximal tubular cells undergo necroptosis, they release endogenous molecules like damage-associated molecular patterns (DAMPs), which activate downstream inflammatory signaling pathways such as Toll-like receptor (TLR) signaling, thus initiating strong inflammatory reactions [155, 158, 159]. Within necroinflammation, macrophages emerge as crucial inflammatory cells capable of activation and polarization into proinflammatory macrophages. In ischemic AKI, the recruitment of proinflammatory macrophages significantly escalates within the initial 48 h [34]. Membranous Toll-like receptors (TLRs) can collaborate with NLRP3 to detect danger signals from necrotic tubular epithelial cells in injured kidneys, activating proinflammatory macrophages by assembling the NLRP3 inflammasome [160]. The adapter protein apoptosis-associated speck-like protein (ASC) plays a crucial role in recruiting and activating caspase-1, the effector molecule [161]. Upon receiving signals from pathogen-associated molecular patterns (PAMPs) or damage-associated molecular patterns (DAMPs), pattern recognition receptors (PRRs) on nucleotide-binding oligomerization domain-like receptors (NLRs) or Toll-like receptors (TLRs) trigger the activation of the ASC adapter, leading to the activation of effector caspase-1 or caspase-11. This activation initiates the secretion of mature proinflammatory cytokines and cleavage of gasdermin D (GSDMD), inducing cell pyroptosis [96, 162–165]. Inflammatory caspases, such as caspase-1 and caspase-4/5 (caspase-11 in mice), cleave critical proinflammatory proteins like GSDMD, leading to cell death via pyroptosis [166–168]. Caspase-1/11 cleavage of intracellular protein GSDMD releases its N-terminal fragment (GSDMD-NT), which oligomerizes to form a membrane pore, facilitating the release of interleukin-1β (IL-1β) and IL-18 into the extracellular space [96, 169]. Although the maturation of these inflammatory cytokines renders this cell death pathway highly immunogenic, the cleavage of GSDMD allows the assembly of a well-defined transmembrane pore, resulting in plasma membrane rupture [170, 171] (see Fig. 2). While the role of pyroptosis in macrophages is well understood, its pathophysiological significance in overall organ damage remains speculative [172]. Although some studies have suggested a role for pyroptosis in acute kidney injury (AKI) [173], several factors should be considered. Firstly, the components crucial for assembling pyroptosis-inducing inflammasomes are minimally expressed by tubular cells. Additionally, the use of lower ischemia doses before reperfusion in kidney ischemia-reperfusion injury (IRI) models might introduce significant artifacts. Furthermore, while antibodies against the N-terminal cleaved fragment of GSDMD are available for immunohistochemistry of human kidney biopsies, gsdmd-deficient mice have not been employed in commonly used AKI models. Therefore, the current evidence is deemed insufficient to establish pyroptosis as a significant contributor to renal tubular necrosis. Nevertheless, the exploration of pyroptosis in AKI remains limited, and existing findings are contentious [173–175]. In the context of AKI and chronic kidney disease (CKD), GSDMD expression dynamics and its implications vary. During the initial 84 h after ischemia-reperfusion injury (IRI) in mice, the expression of GSDMD protein in whole kidney lysates notably rises, especially in the peritubular compartment, but remains undetectable in isolated kidney tubules [176]. Studies on GSDMD-deficient mice indicate heightened vulnerability to injury in both IRI and cisplatin-induced acute kidney injury (AKI) models. Conversely, other research suggests that either genetic deficiency of GSDMD or inhibition of the NLRP3 inflammasome might offer protection in AKI models [173, 174, 177]. Importantly, despite in vivo data post-IRI and single-cell analysis, GSDMD expression hasn’t been observed in tubular epithelial cells following murine kidney IRI [176, 178]. In CKD, the release of double-stranded DNA (dsDNA) from necrotic cells activates the absent of melanoma 2 (AIM2) inflammasome, leading to GSDMD cleavage and inflammatory cell death via pyroptosis. Aim2 deficiency has been linked to massive macrophage accumulation, delayed functional recovery, and perpetuation of fibrosis in the kidney, with kidney macrophages undergoing swift pyroptosis in response to dsDNA [179].

NLRP3 is prominently present in macrophages and tubular epithelial cells, both playing crucial roles in chronic kidney disease (CKD) development. Notably, heightened levels of NLRP3 expression have been identified in kidney biopsies taken from patients with both acute AKI and CKD [180]. Furthermore, in a mouse model of CKD involving nephrectomy and deoxycorticosterone acetate (DOCA) treatment, there was an observed increase in NLRP3 expression [181]. Studies utilizing genetic deficiencies in NLRP3, ASC, or caspase-1 have demonstrated significant protection against renal inflammation, damage, and dysfunction induced by various factors, such as unilateral ureteral obstruction (UUO), 5/6 nephrectomy, crystal nephropathy, or cisplatin-induced kidney injury [8, 182, 183]. Activation of inflammasomes exacerbates kidney damage, whereas inhibition of specific inflammasome signaling pathways often mitigates kidney injury. Hence, targeting the inflammasome holds promise as a therapeutic approach for renal diseases, potentially leading to the development of innovative and efficient treatments. Research has shown the presence of IL-18 and caspase-1 in renal tubular epithelial cells, as well as in patients diagnosed with CKD [184, 185]. In a model of UUO, there was an observed increase in caspase-1, IL-1β, and IL-18 expression, leading to NLRP3 activation. Conversely, in mice with a knockout of the NLRP3 gene, reduced tubular injury and fibrosis were observed following UUO [180]. Knockouts of caspase-1 or NLRP3 result in the attenuation of acute kidney injury induced by ischemia-reperfusion injury [186, 187]. Activation of the NLRP3 inflammasome leads to the release of proinflammatory cytokines such as IL-1β and IL-18, primarily by renal mononuclear phagocytes. However, these cytokines are also released by renal parenchymal cells, including podocytes. Podocytes derived from renal biopsy tissues and urine samples have exhibited the expression of NLRP3, IL-1β, and caspase-1 in patients with class IV and V lupus nephritis [188]. This activation serves as a trigger for renal inflammation in chronic kidney disease (CKD). In the context of an animal model involving UUO, multiple studies have demonstrated that NLRP3 inflammasome-mediated renal inflammation contributes to the progression of CKD [180, 189]. According to reports, Nlrp3-deficient mice exhibited reduced inflammation, fibrosis, and tubular damage following UUO, associated with diminished caspase-1 activation and maturation of IL-1β and IL-18. [13, 180, 189]. According to current research, Nlrp3 absence lessens renal inflammation and ferroptosis, which lessens LPS-induced S-AKI [13]. Interestingly, additional experiments employing bone marrow chimeras have exposed that NLRP3, present in both nonhematopoietic and hematopoietic cellular sections, developed the inflammation and kidney injury [180]. Moreover, investigations on murine and human tubular epithelial cells have disclosed the involvement of NLRP3 in TGF-b signaling. Wang et al., detected a significant decrease in TGF-β-induced expression of MMP-9 and a-SMA in murine cells lacking NLRP3 [190]. Additionally, they noted in human embryonic kidney cells, increased Smad3 phosphorylation and activity upon overexpression of NLRP3. The activation of the inflammasome intensifies the inflammatory response in macrophages, influencing their interaction with other immune and kidney parenchymal cells [191]. In IgA nephropathy mice, IgA immune complexes activate NLRP3 inflammasomes in macrophages, leading to mitochondrial integrity loss and mitochondrial ROS production [143]. In mice with unilateral ureteral obstruction (UUO), the extent of renal fibrosis aligns with the infiltration of M1 macrophages115, which is associated with elevated expression and activation of NLRP3 [180]. The kidney was protected from inflammation and fibrosis induced by adenine and calcium oxalate when NLRP3 was pharmacologically inhibited using MCC950 and beta-hydroxybutyrate, despite exhibiting similar crystal deposition as untreated mice [8, 192, 193]. Systemic NLRP3 inflammasome activation appears pivotal in the development and progression of diabetic nephropathy. Nlrp3 Deficiency Mitigates Acute Kidney Injury Induced by Lipopolysaccharide through the inhibition of renal inflammation and ferroptosis in mice [194]. NLRP3 knockout mice exhibited suppression of diabetic nephropathy in both type 1 and type 2 diabetes by blocking NLRP3-mediated mitochondrial ROS generation [10, 195]. After undergoing unilateral ureteral obstruction (UUO), mice lacking NLRP3 demonstrated decreases in tubular apoptosis, inflammation, and fibrosis [133, 180, 196]. A study revealed that both mRNA and protein expressions of NLRP3 and AIM2 were detected in renal biopsy samples obtained from patients with kidney disease [62, 180, 197, 198]. Consistent with findings in murine models of CKD, there was a direct correlation noted between NLRP3 mRNA expression levels in kidney biopsy specimens and serum creatinine levels in a small group of CKD patients [180]. Additionally, kidney biopsy samples obtained from patients diagnosed with crescentic glomerulonephritis, IgA nephropathy, lupus nephritis, focal segmental glomerulosclerosis, membranous nephropathy, acute tubular injury, and hypertensive or vascular nephrosclerosis displayed heightened NLRP3 mRNA levels compared to those from healthy kidneys. These findings indicate the involvement of NLRP3 in various kidney disorders [49, 180].

## Therapeutic potential targeting macrophages and its signaling agent in renal diseases

Considering that macrophages perform both initiating and moderating functions in diseases, current investigations are delving into treatment approaches aimed at diminishing either the pro-inflammatory or pro-fibrotic functions of macrophages. Alternatively, there is a focus on amplifying the capabilities of anti-inflammatory, anti-fibrotic, pro-resolving, or pro-wound healing macrophages. Various approaches have been utilized, spanning from monoclonal antibodies, small molecule inhibitors, RNA interference, and the delivery of microvesicles, to employing macrophages as a form of cell-based therapy. Strategies directed at influencing chemokines or chemokine receptors responsible for recruiting monocytes and macrophages have emerged as potential therapeutic objectives [199–202]. For instance, blocking CCL2 or its receptor CCR2, an important monocyte chemoattractant, has demonstrated protective effects in models of renal inflammation and fibrosis [203–207]. The use of a CCR2 antagonist has demonstrated efficacy in halting fibrosis progression across diverse preclinical models [208–210]. These promising findings prompted the first clinical trials involving a selective CCR2 antagonist, CCX140-B. The results indicated promise, as evidenced by a decrease in proteinuria among patients with diabetic nephropathy already receiving renin-angiotensin blockade [211]. In vitro, M2 macrophages sub-types such as M2a and M2c exhibit anti-inflammatory properties and suppress renal injury [45, 212]. However, the modulation of in vitro macrophages to become fibrolytic for reducing fibrosis remains unexplored. Another therapeutic approach for treating kidney disease involves the in vivo modulation of macrophages [213]. Notably, in vivo induction of anti-inflammatory macrophages through IL-25 has proven effective in lessening kidney injury in cases of Adriamycin nephropathy [32]. Administering genetically altered macrophages that express heme-oxygenase-1 (HO1) offered kidney protection to mice undergoing ischemia-reperfusion injury [214]. Additionally, M2 macrophages induced by Netrin-1 demonstrated anti-inflammatory effects, safeguarding against renal injury in ischemia-reperfusion injury mice [215]. Despite the increasing recognition of the protective role of anti-inflammatory M2 macrophages, their profibrotic effects pose a significant challenge for therapeutic use. M2 macrophages release substantial amounts of TGF-β, suppressing inflammation while promoting kidney fibrosis [53]. Unlike the ablation of inflammatory M1 macrophages, the removal of anti-inflammatory and reparative M2 macrophages has shown promise in reducing kidney fibrosis [93]. While inflammation is a crucial factor in fibrosis, the profibrotic pathways activated by M2 macrophages extend beyond inflammation. A promising approach to improve the effectiveness of treating kidney diseases involves focusing on specific functional phenotypes of macrophages and key elements within various profibrotic signaling pathways. In the obstructive nephropathy model, inhibiting β-catenin/TCF and promoting β-catenin/Foxo in the Wnt and TGF-β signaling pathways of bone marrow-derived macrophages proved effective [216]. Redirecting β-catenin binding from TCF to Foxo resulted in reduced inflammatory cytokine production by bone marrow-derived macrophages, altered the fate of MMT macrophages, and provided protection against kidney fibrosis [103]. Targeting macrophage activation and infiltration offers a promising approach to preventing renal fibrosis [217]. Deleting macrophage TGF-βRII, for example, has been found to inhibit macrophage infiltration and renal fibrosis following AKI [218]. Inhibiting NF-kB signaling through methods such as antisense oligonucleotides or IkB, its natural inhibitor, not only suppresses classical macrophage activation but also promotes anti-inflammatory macrophages, thus mitigating kidney injury [70]. Additionally, interventions like Quercetin treatment or Wnt5a inhibition have demonstrated efficacy in blocking macrophage infiltration and M2 polarization, thereby preventing ECM production and interstitial fibrosis through a TGF-β1/Smad-dependent mechanism [219]. Syk kinase, known for its pro-inflammatory action, has been implicated in macrophage activation during rapidly progressive glomerulonephritis in humans [220, 221]. Syk kinase inhibitors have shown benefits in various preclinical models, including ischemia-reperfusion injury, ureteric obstruction, vasculitis, glomerulonephritis, and allograft rejection [221–223]. Notably, Syk inhibitors like Fostamatinib are currently undergoing phase 2 trials for IgA nephropathy, with the ongoing FOSTAMR Trial exploring the efficacy of fostamatinib in chronic active antibody-mediated rejection [224]. An IL-1R1 antagonist, anakinra, has demonstrated nephropathy prevention in diabetic mice [10]. Similarly, treatment with anti-IL-1b antibodies attenuated progressive kidney function loss and preserved podocytes in diabetic db/db mice [225]. Research involving humans has demonstrated positive outcomes, such as enhanced vascular endothelial function in chronic kidney disease patients who do not require dialysis, following a 12-week treatment with the IL-1 inhibitor rilonacept [226]. Clinical trials investigating canakinumab, an antibody targeting IL-1β, noted decreased rates of cardiovascular events in atherosclerosis patients with chronic kidney disease (CKD) without impacting renal function [227]. Conversely, a clinical trial involving gevokizumab, another IL-1β antibody, in diabetic kidney disease was terminated prematurely [228]. Despite promising preclinical findings, the use of TNF-α targeting for renal protection in human kidney disease remains controversial. A trial of infliximab in lupus nephritis failed during recruitment. However, TNF-α monoclonal antibodies are commonly used to treat conditions like rheumatoid arthritis, ankylosing spondylitis, or psoriasis, with varied effects on kidney function. While some reports suggest no harmful effects or a slower decline in renal function with TNF-α inhibitors in CKD patients with rheumatoid arthritis, instances of acute kidney injury (AKI), focal segmental glomerulosclerosis, or IgA nephropathy have been reported in patients with ankylosing spondylitis, rheumatoid arthritis, or inflammatory bowel disease treated with TNF-α inhibitors [53, 229–233]. Extensive research has been conducted on miRNA expression profiles in both human and murine macrophages. Studies have identified miR-9, miR-127, miR-155, and miR-125b as promoters of M1 polarization, while miR-124, miR-223, miR-34a, let-7c, miR-132, miR-146a, and miR-125a-5p induce the M2 phenotype in both species [147, 234].

Research conducted in both animal models and humans has demonstrated hyperactivation of the NLRP3 inflammasome in peripheral blood mononuclear cells (PBMCs) and kidney tissue, including podocytes and tubular cells, in individuals with systemic lupus erythematosus (SLE) [188, 235, 236]. Therefore, targeting this pathway appears to be a promising approach for managing lupus nephritis (LN) activity. However, contrasting findings from other studies suggest that NLRP3 inflammasome expression is reduced in the PBMCs of SLE patients and is inversely associated with disease severity [222, 237, 238]. Strategies focused on NLRP3 inflammasomes have primarily targeted downstream proteins such as IL-1β and caspase-1. Anakinra, a recombinant IL-1Ra, has demonstrated effectiveness in managing gout flares among patients with advanced chronic kidney disease (CKD) [239]. Canakinumab, an anti-IL-1β antibody, has significantly lowered the occurrence of major cardiovascular events in CKD patients. Belnacasan, a selective caspase-1 inhibitor, has shown a reduction in fibrosis formation in mice with unilateral ureteral obstruction (UUO) [62]. Activation of NLRP3 by uric acid in macrophages led to heightened tubular NF-κB levels, linked to tubulointerstitial fibrosis and macrophage infiltration in diabetic kidneys. Urate-lowering agents such as Febuxostat and allopurinol not only inhibit Toll-like receptor (TLR) and NLRP3 inflammasome activation but also prevent subsequent M1 polarization. These drugs hold promise for treating chronic kidney disease (CKD) and its complications [53]. Several clinical studies have investigated the effect of allopurinol, a therapy for reducing urate levels, on the progression of kidney disease in clinical settings. One study found no significant difference in the change in estimated glomerular filtration rate (eGFR) from baseline between the allopurinol and control groups [240]. Conversely, larger clinical trials reported that allopurinol did not decelerate the decline in eGFR compared to control groups in patients with chronic kidney diseases [241, 242]. Clinical trials have confirmed that inhibiting the NLRP3 inflammasome signaling pathway often mitigates renal injury [243]. However, the use of colchicine therapy, which requires high concentrations to inhibit the NLRP3 inflammasome, may be limited in patients with kidney dysfunction due to increased drug half-life and the risk of colchicine toxicity [244]. Astragaloside IV has shown protective effects against cisplatin-induced kidney injury by inducing autophagy and suppressing the NF-κB signaling pathway, consequently downregulating the expression of the NLRP3 inflammasome [245]. Anisodamine has demonstrated a protective role in renal ischemia-reperfusion injury by inhibiting endoplasmic reticulum stress associated with thioredoxin-interaction protein (TXNIP)/NLRP3 inflammasome [246]. The effectiveness of inhibitors targeting caspase 1 has been studied in animal models of kidney disease. Ac-YVAD-cmk, a specific and irreversible caspase 1 inhibitor, has demonstrated effectiveness in acute kidney injury (AKI) models in rodents [247]. VX-765 (belynacasan), an orally administered selective caspase 1 inhibitor, decreased the expression of fibrosis markers in mice subjected to unilateral ureteral obstruction [248]. Ongoing human trials are investigating VX-765 in conditions like psoriasis and treatment-resistant partial epilepsy [249, 250]. The peptide inhibitor Ac-FLTD-cmk, derived from GSDMD, hinders GSDMD cleavage by directly binding to the catalytic region of caspase 1, caspase 4, caspase 5, and caspase 11, thereby diminishing pyroptosis. Disulfiram and BAY 11-7082 modify a conserved cysteine in GSDMD, thus preventing GSDMD pore formation. Disulfiram, commonly prescribed for alcohol addiction, requires further investigation regarding its efficacy in experimental and human kidney diseases. MCC950, a specific NLRP3 inflammasome blocker, has exhibited reductions in IL-1β, IL-18 production, and fibrosis in mice with crystal-induced nephropathy [62]. MCC950 was found to have renal protective effects in the db/db model of diabetic nephropathy [12, 251]. However, in contrast to these findings, MCC950 did not provide protection against kidney injury in diabetic apolipoprotein E-deficient mice; instead, it exacerbated renal inflammation and injury [252]. The reasons behind these contradictory results remain unclear [252, 253]. Hederasaponin C, a natural product, effectively inhibits lipopolysaccharide (LPS)-induced acute kidney injury in mice by specifically targeting Toll-like receptor 4 (TLR4) and modulating the phosphatidylinositol 4,5-bisphosphate (PIP2)/NF-κB/NLRP3 signaling pathway [254]. Spermidine, another natural molecule, protects against kidney injury by downregulating NLRP3 inflammasome activation and IL-1β production in renal macrophages [255]. Fisetin, a flavonoid, ameliorates renal fibrosis in the UUO mice model by inhibiting Smad3 phosphorylation [256]. Furthermore, anti-fibrotic treatments such as pirfenidone and FG3019, which target transforming growth factor-beta (TGF-β) and connective tissue growth factor, and are currently undergoing clinical trials, exhibit compelling potential for clinical applications [257]. Additional therapeutic targets against macrophages, NLRP3 inflammasomes, and their downstream molecules are summarized in Table 1.

**Table 1:** Therapeutic strategies and inhibitors targeting macrophages and their signaling agent in renal diseases.

S. No.	Inhibitor/Therapeutics strategies	Target/Mechanism of Action	Reference
1	Macrophages induced with TGF-β, IL-4, IL-13	Exhibit protection in renal damage in murine Adriamycin nephropathy model	[1]
2	Macrophage TGF-βRII deletion	Inhibition of macrophage infiltration and renal fibrosis after AKI	[218]
3	Adoptive transfer of MINCLE silenced macrophages	Protected the kidney in the cisplatin-induced AKI model	[42]
4	interleukin (IL)-25	Polarize M1 to M2 and decrease kidney ischemic injury	[258]
5	Infusion of IL-10 overexpressing macrophages	Protected ischemia injury in an IRI model	[259]
6	Liposome clodronate	Reduced kidney injury by suppressing inflammatory and profibrotic cytokines	[71]
7	miR-30c-5p agomir	Inhibit IRF-1, polarize M1 to M2 and decrease kidney ischemic injury	[32, 72]
8	Iridoid monoterpenoid	Inhibiting kidney macrophages infiltration and activation through MCP-1/CCR2 signaling pathway	[260]
9	MCC950	NLRP3 inflammasomes blockers, reduced kidney fibrosis by reducing IL-1β and IL-18 production	[62]
10	Anakinra	Antagonist to IL-1R; blocking IL-1a and IL-1b	[239, 261]
11	BAY 11-7082	NF-κB and NLRP3 inhibitor, Inhibit GSDMD pore formation	[116]
12	Ac-FLTD-cmk	Inhibits GSDMD cleavage by binding to the catalytic site of caspase-1, 4, 5 and 11and reduced pyroptosis	[262]
13	Tocilizumab	Anti-IL-6 receptor antibody used for under investigation in CKD	[263]
14	TAK-242	TLR-4 inhibitor, ameliorate progressive renal fibrosis in animal model	[264]
15	Belnacasan	Caspase-1 inhibitor reduced fibrosis formation in UUO mice model	[62]
16	FG-3019	Anti-CTGF; CTGF is a downstream effector of TGF-β, it could trigger fibroblast activation and ECM accumulation	[265]
17	Hydroxychloroquine (HCQ)	Attenuated renal fibrosis by inhibiting macrophage activation and MAPK signaling pathways	[266]
18	NBD peptide	NF-κB; Decreases histological lesions, inflammation and fibrosis.	[267]
19	Pirfenidone	Anti-TGF-β; decrease fibrosis in DKD mice model	[268]
20	Clopidogrel	Inhibiting TGF-b/Smad3-mediated MMT by targeting P2Y12 and reducing progressive renal fibrosis	[213]
21	Glibenclamide	K+ channel; ATP sensitive K+ channel inhibitors; Chronic kidney disease	[269]

## Conclusion and future direction

In summary, macrophages play crucial roles in immune surveillance and maintaining kidney homeostasis. They play an active role in advancing renal inflammation, injury, fibrosis, and damage, as well as in the resolution of these conditions. Following renal injury, macrophages adopt distinct phenotypes in response to changes in the kidney’s microenvironment during diseases. M1 macrophages induce renal injury through inflammatory actions, while M2 macrophages contribute to inflammation resolution and damage repair by releasing anti-inflammatory cytokines and the tissue repair mediator TGF-β. However, uncontrolled secretion of TGF-β in chronic kidney disease leads to renal fibrosis. Renal macrophages express inflammasomes like NLRP3, which can detect DAMPs/PAMPs released from injured kidney tissues, activating the NLRP3 inflammasome complex through the NF-κB signaling pathway. This complex triggers caspase-1 activation, leading to the production of mature IL-1β and IL-18 proinflammatory cytokines and cleavage of GSDMD. Consequently, M1 macrophage polarization ensues, ultimately causing renal inflammation, pyroptosis, and kidney fibrosis. Potential therapeutic targets for kidney diseases involve reducing M1 polarization and inducing M2 polarization. Inhibitors of TLRs and NLRP3 inflammasomes, along with their signaling molecules, show promise as therapeutic targets. Additionally, inhibiting macrophage recruitment, proliferation, and transition is crucial. Therefore, targeting macrophage signaling pathways presents a novel therapeutic strategy for treating kidney injury and fibrosis.

Numerous kidney diseases involve crucial contributions from M1 and M2 macrophages. Under normal physiological circumstances, tissue-resident renal macrophages originate from three sources—Yolk sac, Fetal liver, and bone marrow each exhibiting distinct immunological characteristics. The mechanisms through which renal macrophages from diverse origins differentiate into M1 or M2, and vice versa, during disease conditions remain largely unexplored. The role of NLRP3 inflammasomes in different types of renal macrophages in kidney disease remains to be explored. Therefore, studies using specific renal macrophage cell types and NLRP3 inflammasomes-related signaling protein knockout mice for kidney disease models are needed in the future.
